# Applicability of PROACTIVE recommendations on CBRNe risks and threats to passenger rail and metro sectors

**DOI:** 10.1007/s12198-023-00263-3

**Published:** 2023-06-03

**Authors:** Laura Petersen, Grigore M. Havârneanu, Andreas Arnold, Danielle Carbon, Thomas Görgen, Alan Gavel, Tomáš Kroupa, Daria Kardel

**Affiliations:** 1 International Union of Railways (UIC), 16 rue Jean Rey, Paris, 75015 France; 2 Deutsche Hochschule der Polizei (DHPol), Zum Roten Berge 18-24, D-48165 Münster, Germany; 3 Population Protection Institute, Na Lužci 204, Lázně Bohdaneč, 533 41 Czech Republic

**Keywords:** Railway security, CBRNe, Terrorism, Transport security, Vulnerable groups

## Abstract

Passenger rail and metro sectors are no stranger to malicious or unintentional Chemical, Biological, Radiological, Nuclear and explosive (CBRNe) incidents. Over the last years, the EU H2020 Project PROACTIVE has developed recommendations based on a multimethodological approach which involved questionnaires, interviews and workshops with CBRNe practitioners and security experts. However, these recommendations are geared towards specialised first responders. With a particular focus on rail and metro, this paper examines the answers collected from railway and metro security experts during these research activities to see at which level the PROACTIVE recommendations are fit for these sectors. The results show that some of the generic PROACTIVE recommendations for first responders are already being applied. For example, the recommendations regarding inter-agency collaboration appear to already be put in place. In contrast, other recommendations such as the ones on crisis communication and the inclusion of vulnerable groups in preparedness actions could be applicable since important gaps are present. We discuss the major gaps and how they could be filled in the future.

## Introduction

### CBRNe threats in the public transportation environment

Passenger rail and metro sectors are no stranger to Chemical, Biological, Radiological, Nuclear and explosive (CBRNe) incidents, whether they be malicious or unintentional. As of 17 February 2023, the Global Terrorism Database (GTD) lists 2,213 incidents with the search term “rail” (between 1970 and 2020), of which four have used a chemical weapon and 1,758 used explosives (University of Maryland, 2022). The 1995 Tokyo subway Sarin nerve agent attack is an iconic example of a chemical attack in the rail environment. Sarin vapor was intentionally spread through trains and stations. This led to massive confusion at the start of the attacks as people were unaware of what was causing the injuries (OPCW, 2020), demonstrating the invisible and unique nature of some CBRNe threats. As indicated in the GTD, terrorists also target railways and metros with Improvised Explosive Devices (IEDs). Some recent examples are the 2016 Brussels and 2017 Saint-Petersburg attacks, which led to many deaths and casualties (Shvetsov and Shvetsova 2017). Highly toxic radioactive materials are already known to have been used as a means to enhance the impact of an IED (Clutterbuck 2014), resulting in what is known as a ‘dirty bomb.’ While this has not yet occurred on railway premises, there is a growing interest from railway operators and infrastructure managers to prepare against these types of threats (Project SHERPA 2019). The advent of Covid-19 also brought many questions about the preparedness of the sector to biological risks and threats. At the beginning of the pandemic, authorities believed that public transport would be a high-risk virus transmission space. This was later shown to not be the case thanks to the use of masks and the respect of other hygiene measures put in place by the railways during this crisis (UIC, 2021). However, the Council of Europe’s Committee on Counter-Terrorism (CDCT) has suggested that this pandemic could be followed by an increase in the use of biological weapons by terrorists (Council of Europe, 2020), and with a sudden release of a biological agent, such measures would most likely not be in place.

These examples show that the use of CBRNe material to carry out a major criminal or terrorist attack is a serious threat for railway and metro stakeholders. Railway premises and rolling stock, already acknowledged as soft targets for terrorism (Strandberg 2013), are potentially conducive for a CBRNe attack as they provide a favourable environment for spread (e.g., the prevalence of closed spaces, the use of ventilation systems, prevalence of tunnels or underground stations and the large number of people present at stations and onboard trains).

Unintentional CBRNe incidents could also impact the rail sector, such as an accident occurring on a freight train carrying hazardous materials, in particular if an incident with dangerous goods were to occur in the vicinity of a train station or densely populated area. This is demonstrated by the recent 2023 Ohio, USA derailment of a train with 10 cars carrying hazardous substances, which led to multiple small explosions, an evacuation of the local population within a 1 mile radius of the incident, and a planned controlled release of the substance (Romero and Lenthang 2023). While Strandh (2017) showed that rail authorities and rail operators do not have the same mandate in crises as the first responders do, if such an event were to take place on a train or in a station, railway staff would be expected to play a role. For example, during the initial phase of response and before first responders (e.g., firefighters, police) could arrive, frontline and/or security staff would be the first point of contact for those affected (Havârneanu et al. 2022). Furthermore, once the specialised practitioners arrived, railway staff could further act as a supporting partner by, for example, assisting passengers with evacuation (ibid.). Despite these facts, very few guidance documents on CBRNe preparedness and response exist for the rail and metro sectors.

### Vulnerability, accessibility and CBRNe

The concept of vulnerability is often used to describe a characteristic which affects a person or group’s “capacity to anticipate, cope with, resist, and recover from the impact” of a crisis or disaster (Chen et al. 2009). Vulnerable groups may include children, older persons, pregnant women, persons with disabilities, chronic medical disorders or addiction and their carers, institutionalized individuals, ethnic minorities, persons with limited proficiency of the respective national languages or with restrictions regarding use of transportation. According to WHO (2011), around 15% of the world’s population was estimated to live with some form of disability in 2011, a number which was predicted to and certainly has increased due to a growth in the aging population and an increase in chronic diseases (e.g., diabetes, mental illness). Therefore persons with disabilities should be expected to make up part of the population of affected persons in the case of a CBRNe incident. However, simply having a disability does not necessarily make one vulnerable. Indeed, vulnerability should not be seen as a static characteristic. “An individual is not defined as vulnerable by the nature of their vulnerability, but by their personal circumstances at the time of the emergency. […].” (ISO 22395:2018). This goes hand in hand with the concept of “situational disability”, which states that circumstances surrounding a disaster may lead to temporary impairments, such as noise and smoke temporarily reducing hearing and vision among those present, or high levels of anxiety leading to mental impairments (Gjøsæter et al. 2019). Considering situational disability and the fact that public awareness of CBRNe incidents is low (Hall et al. 2020), it seems fair to assume that everyone is vulnerable in a CBRNe incident. Despite this, a recent review of 95 CBRNe guidance documents found that only 33 addressed the management of vulnerable groups and for those that did, little-to-no specific detail was given about how best to achieve this management (ibid.).

### The PROACTIVE project

The EU H2020 Project PROACTIVE (PReparedness against CBRNE threats through cOmmon Approaches between security praCTItioners and the VulnerablE civil society) aims to enhance societal CBRNe preparedness and response through a better harmonisation of Standard Operating Procedures (SOPs) and articulation of the needs of vulnerable groups. It undertakes these efforts together with its Practitioner Stakeholder Advisory Board (PSAB), which is made up of different kinds of first responders, railway and metro representatives, and other categories of practitioners.

The PROACTIVE project has developed a set of ten recommendations on how to close the gap between current practice and the challenges related to CBRNe preparedness and response (Arnold et al. 2021). These recommendations are based on two main data sources, collected and analysed through social science and humanities research methods. The first source is a questionnaire with 405 Law Enforcement Agencies (LEAs), first responders, and other relevant practitioner categories. The second source is interviews with 48 CBRNe experts to identify common approaches for first responders in assessing CBRNe threats and the protocols and tools used to help citizens (Arnold et al. 2021). Within this paper, a third data source is examined, based on a workshop which PROACTIVE organised to understand practitioners’ views on the SOPs they use (Gavel et al. 2021). All of these data sources used convenience sampling as a collection method.

## Method

### Aim of the current paper

While the recommendations PROACTIVE produced are mainly aimed at specialised first responders (e.g., LEAs, fire fighters, emergency medical services), rail and metro representatives from the PSAB also participated in the research. As such, with a particular focus on rail and metro transport, this paper looks more closely at the data provided by these experts during these research activities to see at which level the ten PROACTIVE recommendations are fit for the rail and metro sectors.

This paper examines exclusively the rail and metro expert responses to three complementary datasets, whereby data was collected using convenience sampling: the questionnaire which was answered by 7 experts, interviews with 4 experts, and a project workshop with an online live poll answered by 3 experts. It then compares the findings to the PROACTIVE recommendations and examines whether or not they are fit for the rail sector based on the combined answers provided by the total sample of 14 railway or metro experts in the three workstreams. All studies received ethics approval from the PROACTIVE Project Ethics Officer.

### Questionnaire on common approaches

Out of a larger questionnaire developed by the project partners with a total of 43 questions (Arnold et al. 2021), responses to 12 questions are considered in this paper (see below). This is mainly due to the nature of the questionnaire, which was heavily focused on first responders, and therefore some questions were not applicable to the public transport sector. The selected questions cover the topics of familiarity with CBRNe and the preparedness of one’s organisation (trainings, staff informational resources), the clarity of roles, communication strategies for CBRNe and the inclusion of vulnerable groups. Question responses were closed and formats included Likert scales, Yes/No and multiple choice with multi select answer options. Some questions were only asked to those respondents who answered positively to the leading question. In total, seven railway experts answered the questionnaire. A quantitative and qualitative analysis of the answers was performed.

1. How familiar are you in general with the topic CBRNe (knowledge about CBRNe)?
2. How often have you been professionally involved in a CBRNe incident throughout your career?
3. How would you assess the level of preparedness of your organisation for a CBRNe incident?
4. Has your organisation established institutional collaboration with public, private and / or social organisations grouping vulnerable groups to tackle CBRNe incidents?
5. Does your organisation have specific SOPs for CBRNe incidents?
  - If yes, which vulnerable groups do the specific CBRNe SOPs (Standard Operating Procedures) take into account?
6. Has your organisation been regularly (i.e. at least once a year) involved in practical/realistic exercises simulating CBRNe incidents in the last ten years?
  - If yes, how valuable do you think these CBRNe exercises have been in preparing your organisation for a CBRNe incident?
7. Are there special - written - cooperation agreements between your organisation and other organisations (LEAs, Fire Brigades, etc.) for major emergencies, which specify the distribution of tasks / cooperation in major emergencies?
  - If yes, how helpful were these agreements for the cooperation during major emergencies?
8. Which information resources does your organisation provide for your personnel to prepare for and to cope with a CBRNe incident?
  - If yes, what substances do they deal with?
  - If yes, how relevant do you think the information resources are in preparing your organisation for a CBRNe incident?
  - If yes, which vulnerable groups do the information resources take into account?
9. Does your organisation have a communication strategy for major emergencies?
  - If yes, how suitable do you think the communication strategy is to respond to CBRNe incidents?
  - If yes, which vulnerable groups does the communication strategy explicitly take into account?
10. Which CBRNe-related information resources does your organisation provide for the public to cope with a CBRNe incident?
  - If yes, how do you assess the effectiveness of the information resources for the public?
11. How do you assess the (assumed) clarity of responsibilities within your organisation during these CBRNe incidents, from very high level to very low level of clarity?
12. How do you assess the (assumed) clarity of responsibilities between the operational forces (e.g. Fire brigades, LEAs, Medical staff) during these CBRNe incidents from very high level to very low level of clarity?

### Interviews

Interviews were carried out with four relevant railway stakeholders and lasted about an hour based on the following interview topics: (Joint) Threat assessment, Legal and policy framework for inter-agency collaboration, (Joint) Training, Evaluation and capacity building, Security measures and Communication with the public. A content analysis of the answers was performed.

### Workshop with online live polling

The workshop focused on the perception of SOPs and used a live online poll with six questions requiring a Yes/No answer and the possibility to leave a comment explaining the answer. Three railway experts participated in the workshop from Canada and from the UK. A content analysis of the answers was performed. The questions can be seen below:

1. Do you find SOPs relating to CBRNe events easy to read and understand?
2. Do SOPs give you the information you need to deal with incidents?
3. Do you find SOPs useful during incidents/ exercises?
4. Do SOPs give you the information you need to communicate with, and manage, members of the public?
5. Do SOPs include enough information relating to the needs of vulnerable groups?
6. Would you want greater standardisation of SOPs across countries?

### Procedure of recommendations analysis and ranking

Based on the combined data from the three workstreams, the 10 recommendations were ranked in order of priority to implement in the rail and metro sectors following a multi criteria scoring system assigned by two independent coders.

The first step in the process was to regroup the data collected by relevance to a given recommendation.

Then, the data was analysed using a quantitative analysis based on two categories, coupled with a qualitative assessment for a third category.

The first category was based on whether or not any data of relevance to the recommendation was found in a given work stream (going from 0 points for no workstreams to 3 points for all three workstreams). The second category was how many participants provided data of relevance to the recommendation (going from 0 participants through to 14 participants). Lastly, the third category was the level of evidence regarding the implementation of a given recommendation, for which a qualitative assessment was conducted by two coders. For each recommendation, the two coders independently assigned points based on the following scale:

- No evidence of awareness or actions regarding the implementation of this recommendation = 0 points.
- Some evidence of awareness or action = 1 point.
- Compelling evidence of actions taken to meet this recommendation = 2 points.

Coders agreed on all point allocations except for two recommendations, where live discussion enabled consensus to be reached.

The number of points assigned on the three categories allowed each recommendation to obtain a total score between 0 and 20, with higher scores suggesting higher evidence of implementation of the recommendation. For example, Recommendation 5 obtained a total score of 3. This score resulted from a low number of workstreams (1), few respondents evoking the topic (2) and a lack of compelling evidence about its implementation based on the qualitative data (0). This total score of 3 therefore suggests that the recommendation currently is not being applied to the sector, reflecting a gap. This is why it has been ranked as high priority to implement (ranking 1).

In contrast, Recommendation 10 obtained a total score of 14, reflecting that all 3 workstreams addressed the topic, 9 participants provided data of relevance, and both coders found compelling evidence of actions having been taken to meet the recommendation in the sector (2). The high score suggests that the gap might be less important. This is why it has been ranked as low priority to implement (ranking 9).

Based on their total score, all recommendations were then ranked from highest priority (lowest total score) to lowest priority (highest total score) to implement.

## Results

### Quantitative and qualitative results from the questionnaire data

#### Responses of transport stakeholders

**Fig. 1:**
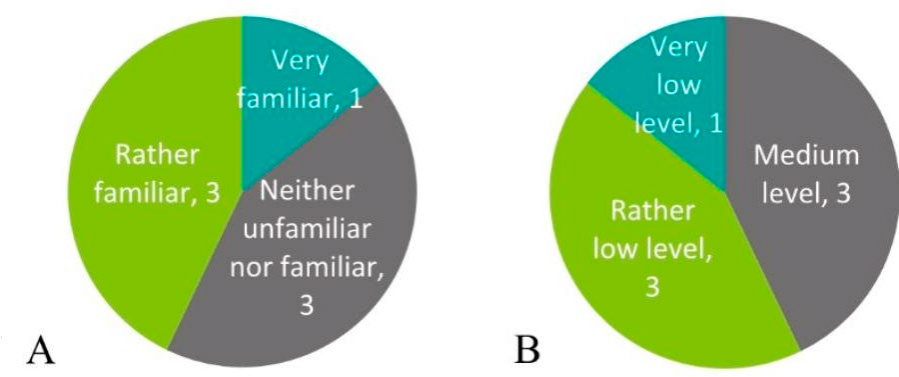
**A**) Organisation’s familiarity with the CBRNe topic in general (questionnaire responses, n = 7). **B**) Organisation’s CBRNe preparedness (questionnaire responses, n = 7)

The questionnaire responses reveal that about half of respondents describe themselves as either very or rather familiar with CBRNe (Fig. 1A), despite only two respondents ever having been involved in an actual CBRNe incident. Respondents do not assess the preparedness level of their organisation as high (Fig. 1B).

**Fig. 2:**
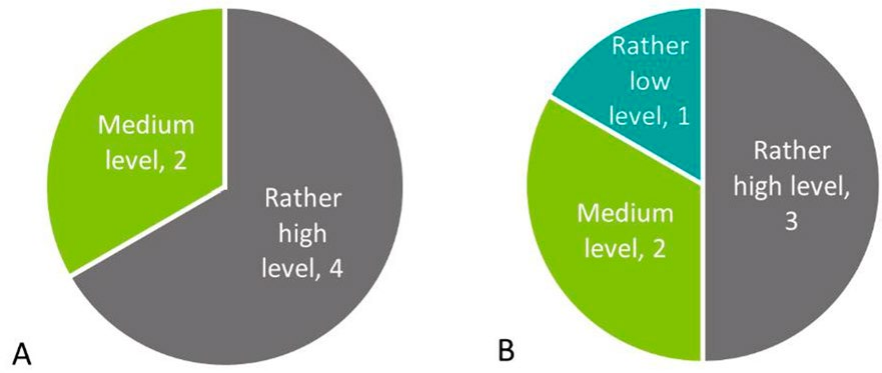
**A**) Clarity of responsibilities within the organisation during CBRNe incidents (n = 6). **B**) Clarity of responsibilities between different organisations during CBRNe incidents (n = 6)

Concerning the clarity of roles during a CBRNe incident, two questions were asked: one focusing on the clarity of roles within one’s own organisation and one focusing on the clarity of roles between different organisations (e.g., fire brigades, LEAs) (Fig. 2). Both questions had six respondents, since one participant decided to skip these questions. For clarity within one’s own organisation, four respondents think the level is rather high whereas two respondents think it is at a medium level. For clarity between organisations, three respondents think the level is rather high, two think it is at a medium level and one thinks it is at a rather low level.

When asked if they collaborate with civil society organisations representing vulnerable groups in order to tackle CBRNe incidents, three respondents said ‘yes’, two said ‘no’, and one said ‘I don’t know’. Only two respondents confirmed that their organisation has CBRNe specific SOPs. The vulnerable groups those SOPs take into account are hearing impaired people and people with no or insufficient language skills of the national language. Only three respondents state that their organisation participates regularly in a CBRNe training exercise. Of those three, each gave the value a different rating, from extremely valuable, to somewhat valuable and slightly valuable. Of the three, two didn’t know if the trainings included vulnerable groups and one confirmed that they do not. When it comes to written cooperation agreements with first responders, two respondents said ‘yes’, three respondents said ‘no’, and one said ‘I don’t know’. The two respondents who have such agreements found them to be very helpful.

**Fig. 3:**
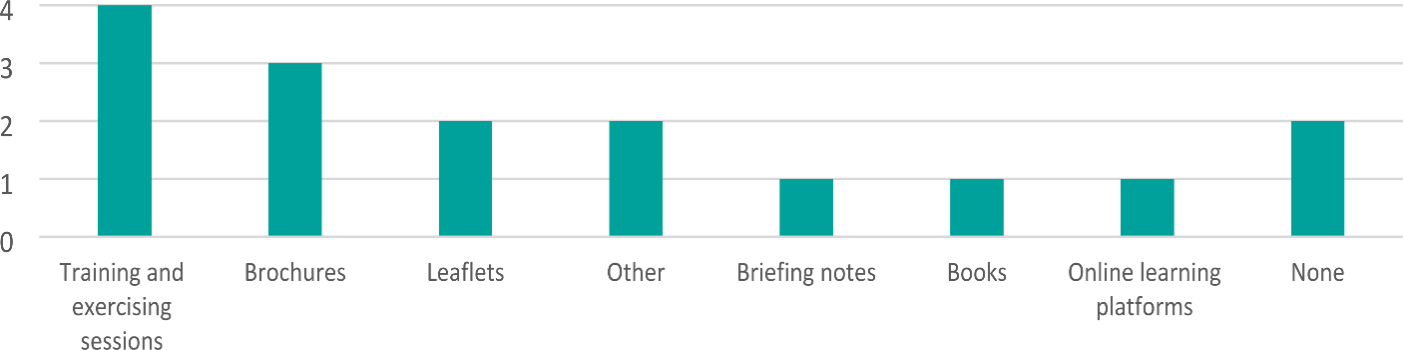
CBRNe information resources provided for staff, multiple choice with multi select answer options (n = 7)

When asked about providing staff with CBRNe information materials, five respondents said that their organisation provide materials in a variety of means, from training exercises to leaflets (Fig. 3). Mostly these materials deal with biological, radiological or nuclear substances. Three respondents found the staff information materials to be very relevant and one respondent found them to be only somewhat relevant. When asked to mention which vulnerable groups are taken into account in staff CBRNe information materials, three respondents did so, but one stated that their organisation’s information materials do not take any vulnerable groups into account. The two respondents whose organisations consider vulnerable groups mentioned the following: children, older persons, people with mobility restrictions, visually impaired people, people with no or insufficient language skills of the national language, and pregnant women.

**Fig. 4:**
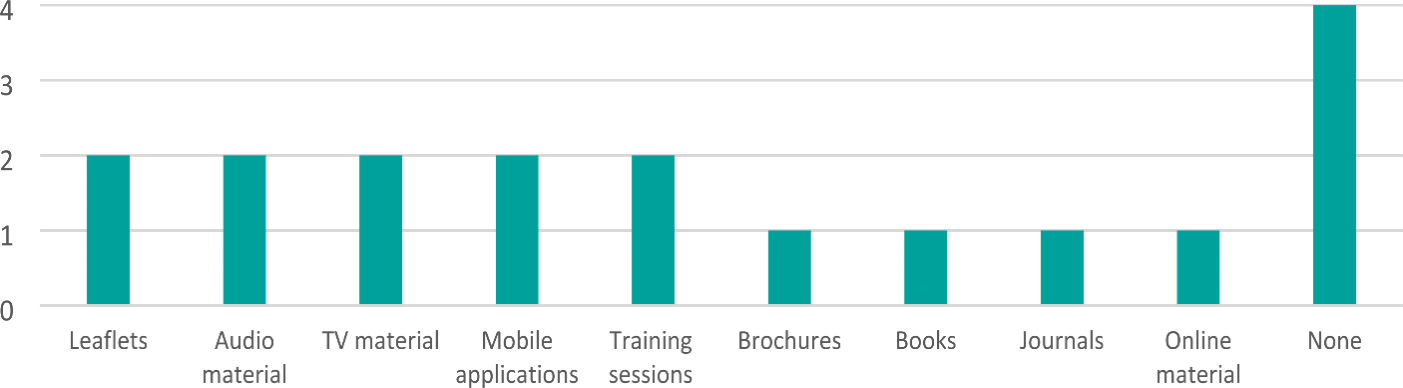
CBRNe information materials resources for the general public, multiple choice with multi select answer options (n = 7)

When asked about strategies for communication with the public, six respondents stated that they have such a strategy and one responded that they didn’t know. However, only one respondent thinks that the communication strategy is very suitable for CBRNe incidents, with three respondents finding their communication strategy only somewhat suitable. When asked to check which vulnerable groups are taken into account in the communication strategy, only two respondents did so. They mentioned the following vulnerable groups: children, older people, people with mobility restrictions, visually impaired people, hearing impaired people, people with no or insufficient language skills of the national language, and pregnant women. When asked about information resources they provide to the general public about CBRNe, three respondents state that their organisations provide such information through a variety of means (Fig. 4). Out of those three, two respondents chose rather high effectiveness for the materials and one preferred to skip the question.

### Qualitative results from the interviews

The following part presents the findings of interviews with railway stakeholders from four different countries: Japan, France, Turkey and Canada.

#### Japan

Japan is one of the first countries in the world to have experienced a chemical attack in a public mass transit setting (the Tokyo metro sarin attack in 1995). To respond to the challenge of large and congested metropolitan areas, the railway and other public transport operators in Japan (around 200 operators, most of them private companies, among which about 30 are major ones) developed very tight cooperation with the first responders divided into small, local areas. They work very closely with the fire department, as explained by the interviewee, “we have 12 branch-offices, and each branch office has to connect, communicate with a lot of fire departments. Each branch will cooperate with its local fire department.” They also have close ties to police, “on local level we collaborate, we cooperate with them [the railway police] for preventing terror attack and a chemical attack, and to rescue the victims of the disaster.” One role of the railway identified by the interviewee is to provide information to the police, “if we have a possible terror [attack], the police organization request to get information from us and we will give the information in time, immediately.” This cooperation also includes training, “We have a lot of drill, practice with … the police and fire department.” Cooperation also exists in the rural and more remote areas, despite their lower security risk.

The long distances between the large metropolitan areas are challenging for the railway companies which operate high-speed trains when preparing special measures in case of a disaster or attack. Preparing measures for evacuation is easily envisaged for metro, as explained “the metro stations are closer (roughly every 2 kilometres) and passengers can evacuate easily. We prepare the evacuation route and we can give a good guide. Drivers, station staff and maintenance engineers will support on evacuation of passengers if we have a terror [attack].” However, such policies are harder to implement in rural areas or on high-speed trains as “high-speed rail has a few stations, from Tokyo to Aomori it takes about four hours. The fastest train will stop at three stations, so most of the time it’s running and is closed.” This means it takes longer for first responders to reach the area.

Concerning communication with passengers and station visitors, the railway operators are open and transparent. They send passengers detailed information via their smartphone App and SMS notification system. They send the same information to the passengers as they do their railway staff. Therefore, nowadays “few passengers go to the station staff and the driver in order to get a personal information but maybe 90% or 95% of the passengers can decide their action from our information.” Furthermore, this availability of information has been found to be “very important to the reduced mobility persons.”

#### France

In France, a metro operator has staff who are trained in CBRNe and can use relevant protective equipment. For example, some train drivers and maintenance personnel are trained to use personal protective equipment and operate in a contaminated area, as explained by the interviewee. In addition, a limited number of staff have a good understanding of the challenges associated with CBRNe incidents. The preparedness process started around 10–12 years ago after some meetings between the transport operator and the French Gendarmerie’s CBRNe cell. This helped the transport operator realize the importance of this threat.

Today there is a national strategy which specifies the roles and responsibilities of various first responders in some CBRNe-related scenarios. However, the metro operator is not directly involved in these since CBRNe procedures (e.g., decontamination) remain clearly outside of their area of responsibility. The metro operator is mainly concerned with the probability of a chemical scenario and its main role is to initially identify and safeguard the incident area, manage evacuation and ensure compliance with the instructions given by the emergency services as soon as they are present.

When it comes to communication with the public about CBRNe, the strategy appears to be to keep silent. As explained by the interviewee, there is a general feeling of “we don’t want to create panic and the simplest way is not to communicate about it.” This means that if an attack similar to what happened in Tokyo in 1995 happened nowadays in France, the interviewee continued, “nobody will say Oh, it’s probably like Tokyo.” That said, ever since the 2015 Paris terrorist attacks, there is communication plan called VIGIPIRATE where it is explained to the public how they should act if a terrorist attack were to occur (SGDSN, 2016). The interviewee therefore suggests, “it could also say that a terrorist attack alert, it’s not just explosives or people with a knife or armed people, it could also be something else [CBRNe].”

#### Turkey

In Turkey, the interviewee, who works for a railway company, focused on the transportation of dangerous goods and hazardous materials. The railway company is public and operates under the authority of the Ministry of Transport. If a disaster occurs in Turkey, the Presidential Disaster Management Office (AFAD) coordinates the response and decides the responsibilities of the different Ministries (e.g. Health, Transport). As such, AFAD is responsible for CBRNe scenarios and this is not the direct responsibility of the railways. When an incident occurs (including CBRNe), the railways are only responsible for evacuation. In addition, there is a civil defence unit in the railway which is involved in response.

According to the interviewee, the Turkish railway company does not have specific CBRNe trainings, however there are informative signs in the company and other facilities which indicate e.g., what to do in case of a nuclear attack. Further, there are well developed trainings on the transport of dangerous goods. In a part of this training program, disasters such as Chernobyl and Fukushima are mentioned. However, there are no specific recommendations for CBRNe attacks.

#### Canada

Canada’s metro and rail stakeholders have past experience with CBRNe. As explained by the interviewee, there was a situation when protestors “were actually targeting areas like the railway or the subway… They were putting these smoke-type bombs within the trains and the rail system, which would cause a huge disruption of the service.”

Canada undergoes a joint threat assessment when it comes to CBRNe and the protocols are shared amongst all stakeholders, including railways. The interviewee views the inter-agency collaboration as “strong.” Indeed, “almost all police agencies and not just police agencies, for example, if we take the railways, if we take the transit and this, they all have very similar protocols, they coordinate and they try to make sure that they are in-sync.” The inter-agency collaboration extends to the national Canadian training exercises, where the railway company is invited to attend. As explained by the interviewee, “for example at my company, we participated in a huge exercise a few years ago … with the emergency medical service, the police, you know, the intelligence units, the investigative units, with real actors, command centres, cameras to observe the events, debriefs and briefs before.”

Further, the interviewee described the recent accessibility policies which passed into law in Canada, obliging railways to consider vulnerable groups in all areas, including CBRNe preparedness and response. They are now viewing all their policies through the lens of accessibility. An example provided by the interviewee is that they “try to embed within an emergency now, within a station and [if] there is a person experiencing homelessness, how we will be able to help that person.”

### Qualitative results from the workshop with online live polling

All three participants in the workshop find CBRNe SOPs easy to read. One participant highlighted that while they have no issues, “someone with limited knowledge might want an annex with pictures or reference.” All three participants agreed that SOPs provide enough information to deal with incidents and that SOPs are useful during both incidents and exercises. The importance in exercising SOPs was agreed, with one respondent suggesting, “As low likelihood / severe impacts, we may not always have enough practice on them, especially for non-specialist units. This has proven very problematic in the 1995 Sarin attack.”

All participants disagreed with the statement that SOPs give them the information needed to communicate and manage the public. Communication strategies tend to cover all crises and, as one participant put it, “the plan should clearly outline the differences between other events and CBRNe incidents, as the later can typically cause wider impacts due to the lack of familiarity of the public with these threats.” Similarly, no respondents think that SOPs include enough information on vulnerable groups. One participant stated that they would like SOPs to address how to overcome the challenges of reaching vulnerable groups and gave the example of homeless people with no identity papers following an R or B attack. Another mentioned that while they “play a major role in providing proper service to disabled people,” it is not in relation to CBRNe and think that SOPs should be “adapted with a lens in viewing them to the needs of vulnerable groups.” The third specified that they would like more information on “visually impaired and other impairments” as well as best practice for meeting the needs of vulnerable groups. Lastly, regarding standardization, all three participants want greater standardization across countries, but insist on the “need to be some leeway to adapt to specific context.” Indeed, one participant said, “take the Mumbai and Paris metro attacks, the UK adapted training packages to these incidents, but what they did might not work here and vice versa.”

## Fit of PROACTIVE recommendations to railway and metro sectors

The results which emerged in this paper from the subgroup of railway and metro experts are grouped to the PROACTIVE recommendations (see Table 1). The scoring and final ranking of the recommendations can be seen in Table 2.

**Table 1:** PROACTIVE Recommendations from Arnold et al. (2021) with data from railway and metro experts collected from the three different data sources

No	Recommendation	Relevant Questionnaire Data (7 participants)	Relevant Interview Data (4 participants)	Relevant Live-Poll Data (3 participants)
**1**	The needs, expectations and challenges in regard to vulnerable members of the civil society should be considered more extensively in CBRNe-related SOPs.	Of the **2** railway stakeholders which state that their organisation has CBRNe SOPs, these only take into account hearing impaired people and people with no or insufficient language skills of the national language.	**None** of the interviewees specifically mentioned SOPs, however **1** interviewee mentioned that policies must now be seen through an accessibility lens and specifically mentioned homeless persons.	**0** respondents think that SOPs include enough information on vulnerable groups.
**2**	Vulnerability should be addressed more intensively in CBRNe-related discussions to raise awareness of the needs, expectations and challenges in regard to vulnerable members of the civil society in CBRNe incidents.	Out of the five respondents who said that their company provides staff materials on CBRNe, **4** of those stated that they included information specific to the following vulnerable groups: children, older persons, people with mobility restrictions, visually impaired people, people with no or insufficient language skills of the national language, and pregnant women.	**2** interviewees mentioned vulnerable groups, one mentioning homeless persons and another mentioning persons with reduced mobility (PRM).	**0** respondents think that SOPs include enough information on vulnerable groups
**3**	More extensive inter-institutional cooperation between organisations involved in CBRNe incidents and CSOs should be sought.	**3** respondents said that their companies collaborate with CSOs on the topic of CBRNe, while 2 said no.	No interviewees mentioned working with CSOs on this topic.	N/A
**4**	An increase in regular CBRNe exercises is desirable to train SOPs, to harmonise procedures, and to increase the level of preparedness and the clarity of responsibilities. Furthermore, inter-agency exercises should be conducted more regularly to create an understanding of the responsibilities of other involved practitioners during a CBRNe incident.	**3** companies participate regularly in CBRNe training exercises and these respondents confirmed to train together with LEAs, Fire Brigades and Medical Staff at least sometimes.	**2** interviewees specifically mentioned being part of CBRNe training exercises	**All (3)** agree that training is important
**5**	An increase in regular CBRNe exercises that involve members of the vulnerable civil society is desirable to train specific SOPs, to adapt relevant procedures, and to increase the level of preparedness.	Of the 3 above, **2** didn’t know if the trainings included vulnerable groups and **1** confirmed that they do not.	Whether or not vulnerable groups were included in the trainings was not discussed.	N/A
6	In particular, the topics of “containment”, “evacuation”, “decontamination” and contact with (vulnerable) citizens should be trained during CBRNe exercises.	*The frequency with which the rail and metro sector include these elements in their training could not be evaluated and as such the applicability of this recommendation remains unknown at this time.*		
**7**	LEAs and first responder organisations should review their SOPs, cooperation agreements, etc., to determine if the documents are clear enough about responsibilities during a major incident (including CBRNe incidents). This includes responsibilities within their own organisation as well as the sharing of responsibilities between the individual organisations (fire brigades, LEAs, etc.) in the event of a major incident.	**6** respondents think that the clarity of roles within their own organisation is at a medium level or higher, and 5 respondents think that the clarity of roles between organisations is at a medium level or higher.	**4** interviewees mentioned that there is coordination between organisations and that their role is clear.	N/A
**8**	Communication before, during and after a CBRNe incident should support the public more effectively to prepare for and to cope with the specifics of a CBRNe incident.	This recommendation would appear to involve both crisis communication and preparedness communication. Concerning crisis communication, while 6 respondents state that they have a strategy, only **1** respondent thinks that the communication strategy is very suitable for CBRNe incidents. Concerning preparedness, only **3** respondents stated that their company provides information materials to the public to help them prepare for an incident, but for those that do, these materials are viewed as effective.	**2** interviewees mentioned communication with the public.	**0** respondents agreed that SOPs give information needed to communicate with, and manage, members of the public.
**9**	The needs of vulnerable groups should be addressed more frequently in communication strategies before, during and after a CBRNe incident. Thereby, first responder organisations should acknowledge and understand the diversity of their audiences prior to a CBRNe event in order to be able to increase the number of those who actually understand their information.	In the questionnaire, only **2** respondents stated that vulnerable groups were taken into account in either communication strategies or preparedness materials. They stated that the following vulnerable groups were taken into account: • Children • Older persons • People with mobility restrictions • People with visually impairments • People with hearing impairments • People with no or insufficient language skills of the national language • Pregnant women This means that the following vulnerable groups listed in the multiple choice answer are not currently taken into account: • People with mental health conditions • Ethnic minorities	**1** respondent specifically mentioned vulnerable groups and informed that they have a legal obligation to consider vulnerable groups’ needs in CBRNe preparedness and response.	Specifics regarding vulnerable groups was not asked, but can be deduced from above that communication strategies also don’t meet the need of vulnerable groups
**10**	There should be a stronger development of systems of joint cooperation. These include joint-threat assessment and joint-coordination centres (see Recommendation 7)	Only **2** respondents in the questionnaire stated that they have written cooperation agreements with first responders	Signs of cooperation are present in Japan, Canada and France since all interviewees reported on the close collaboration between railways and police, firefighters, etc.	**3** respondents would like greater harmonization of SOPs

**Table 2:** Ranking of recommendations from high to low priority based on the total score

Rank	Recommendation	Workstreams score	Respondents Score	Evidence Score	Total Score
**1**	5	1	2	0	**3**
**2**	3	1	3	1	**5**
**3**	1	2	3	1	**6**
**4**	9	2	3	2	**7**
**5**	2	2	6	1	**9**
**6**	8	2	6	1	**9**
**8**	4	3	8	2	**13**
**7**	7	2	10	2	**14**
**9**	10	3	9	2	**14**
**10**	6	N/A	N/A	N/A	N/A

## Discussion

### The CBRNe niche within railways and metros

Despite the low probability high impact nature of CBRNe incidents, railway companies appear to provide information sources for their staff about this topic. Unsurprisingly, the main subtopics communicated about include chemical attacks and explosions since these are the types of attacks which have occurred in the past. Past attacks such as the Tokyo Sarin Attack of 1995 came up multiple times in both the workshop and the interviews, and participants demonstrated a strong desire to apply lessons learned from past events to their own situations. That said, standards which are too rigid are not expected to be useful and should be avoided. Instead, general recommendations and guidance as well as good practices from past events can be used to improve security.

### Recommendations on inclusion of vulnerable groups strongly apply to the rail and metro sectors

It would seem that there is a wide gap regarding the inclusion of vulnerable groups in the area of CBRNe preparedness in the public transport sector. For those metro and rail participants that do have CBRNe SOPs, only two vulnerable groups are found therewithin. Furthermore, railway and metro experts expressed interest in having more information on how to handle such persons during an incident. However, in Canada it seems that a legal obligation has forced them to actively consider vulnerable groups in CBRNe preparedness and response. As such, for those without SOPs, when drafting them, they should make an effort to include information about how to manage vulnerable groups.

While the transport sector does appear to train for CBRNe incidents, vulnerable groups do not seem to be part of such trainings. This is a big gap, especially considering the high likelihood that vulnerable persons may be travelling by rail or metro, and the various accessibility requirements transportation operators face regarding PRMs. Lastly, there would appear to be room for improvement when it comes to working together with CSOs on the topic of CBRNe.

As such, recommendations 5, 3 & 1 are of high priority for application to the rail sector.

### Crisis communication not seen as fit for CBRNe, but addresses well the aspect of vulnerability

There seems to be two different ways of viewing communication about these types of incidents. On the one hand, in Japan communication seems to be rather open about such attacks, while, on the other hand, it would appear that in France communication about CBRNe is much more restricted. While some companies are providing public information materials, others are not. Further, while crisis communication strategies exist, they are not deemed fit for the particularities of a CBRNe attack. Based on these results, it would seem that transportation stakeholders could do more to support the public when preparing for and coping with CBRNe incidents. However, it appears that transport stakeholders already ensure that their communication strategies are understandable by several different vulnerable groups.

When it comes to awareness levels of staff, the fact that most information materials do mention vulnerable groups is a great step into raising awareness of the needs of vulnerable persons in CBRNe. However, the fact that one company doesn’t provide staff materials at all and another one does not have information about vulnerable groups would seem to suggest that more could be done in this realm. Further, it seems that railway respondents would also like more information on vulnerable groups in SOPs.

As such, recommendations 2, 8 & 9 should also be applied to the rail and metro sectors.

### Close cooperation, including training exercises, between rail and metro stakeholders and specialized first responders seen as best practice

Since it seems that the public transport sector in several different countries already engages in essential cooperation with practitioners, including trainings. Even if the cooperation isn’t always in a written or legally binding document, this cooperation can be seen as good practice. Furthermore, roles appear to be well defined both within an organisation and between transportation stakeholders and first responders.

With this in mind, it may be said that recommendations 4, 7 & 10 are already being applied to a certain level in the rail and metro sectors.

## Limitations

The results reported in this paper have several limitations inherent to the research method used and the sample size. The data we used was extracted from different project activities which involved rail and metro respondents, and which were not designed to be necessarily combined. The open nature of the questionnaire with semi-structured questions employing various answer modes and open answers allowed only descriptive and qualitative analyses. The interview questions and the poll conducted during the workshop did not always perfectly match the survey questions, allowing to make just generic links with the overall recommendations. Moreover, very few of the participants in the overall PROACTIVE research streams came from the rail or metro sectors, which led to a small total sample in this study. This is most likely because CBRNe is a niche field, not even necessarily well known by all first responders, and in the transport sector it is even more difficult to find CBRNe experts (this being a niche within a niche). The small sample and the convenience sampling technique that we used in the three workstreams does not allow for these results to be generalised. The findings reported in this paper should therefore be considered within these methodological limitations and the descriptive scope of the study.

Despite these limitations, this paper is one of the first attempts to compile various information streams from the rail and metro sector. This work enabled the collection of valuable data (otherwise very difficult to access) from railway and metro security experts who are generally difficult to reach and have limited time resources for research activities. As a result, the qualitative and quantitative findings are not generalisable, but do provide worthwhile insights into the sector’s approach in preparing for and responding to CBRNe events.

## Conclusion

This paper examines the results relating to the rail and metro sectors from three data sources focused more generally on CBRNe preparedness and response. The paper then examines whether or not the data from the rail and metro experts is in line with the PROACTIVE project recommendations. The paper finds that the recommendations regarding crisis communication and the inclusion of vulnerable groups also hold true for the rail and metro sectors. The paper also finds that the rail and metro sectors are already applying the recommendations of close collaboration between first responders and other actors. Taking into account that this paper is studying the niche of CBRNe within the niche of public transport, it is an important empirical first look into how the railway and metro sectors should approach this low probability high impact threat.

## Data Availability

The qualitative data collected is directly available in the core of this article. The interviewees did not consent to their transcripts being shared. However they did consent to anonymized quotations from the transcripts being used in academic publications.
